# Metals Enhance the Killing of Bacteria by Bacteriophage in Human Blood

**DOI:** 10.1038/s41598-018-20698-2

**Published:** 2018-02-02

**Authors:** Li Ma, Sabrina I. Green, Barbara W. Trautner, Robert F. Ramig, Anthony W. Maresso

**Affiliations:** 1 Molecular Virology and Microbiology Department, Baylor College of Medicine, Houston, TX USA; 2 Michael E. Debakey Veterans Affairs Medical Center, Houston, TX USA; 3 Department of Medicine, Baylor College of Medicine, Houston, TX USA

## Abstract

Multidrug-resistant bacterial pathogens are a major medical concern. *E*. *coli*, particularly the pathotype extraintestinal pathogenic *E*. *coli* (ExPEC), is a leading cause of bloodstream infections. As natural parasites of bacteria, bacteriophages are considered a possible solution to treat patients infected with antibiotic resistant strains of bacteria. However, the development of phage as an anti-infective therapeutic is hampered by limited knowledge of the physiologic factors that influence their properties in complex mammalian environments such as blood. To address this barrier, we tested the ability of phage to kill ExPEC in human blood. Phages are effective at killing ExPEC in conventional media but are substantially restricted in this ability in blood. This phage killing effect is dependent on the levels of free metals and is inhibited by the anticoagulant EDTA. The EDTA-dependent inhibition of ExPEC killing is overcome by exogenous iron, magnesium, and calcium. Metal-enhanced killing of ExPEC by phage was observed for several strains of ExPEC, suggesting a common mechanism. The addition of metals to a murine host infected with ExPEC stimulated a phage-dependent reduction in ExPEC levels. This work defines a role for circulating metals as a major factor that is essential for the phage-based killing of bacteria in blood.

## Introduction

The rate at which new antibiotics are discovered, developed, and approved lags behind the pace at which selective pressures drive the evolution of bacterial resistance to these drugs^1^. Paradigm-changing treatments, or approaches that facilitate their rapid development and safety testing, would be a valuable ally in the fight to combat this growing problem. Bacteriophages are one possible therapeutic solution to the resistance conundrum because of their ability to kill multidrug-resistant bacterial strains^2^, the ease at which they can be isolated and characterized^2,3^, and their rapidly evolvable properties^4^. However, a roadblock to the development of phage as specialized therapeutics has been the dearth of information concerning how phage function in mammals, particularly in the human body. For example, whereas a reasonable level of knowledge is available concerning the basic biology of phage infection of their bacterial hosts in commonly-used laboratory culture conditions, these results do not always translate when assessed in animal models of efficacy^5^. Thus, there are unidentified factors present in mammalian systems that influence the ability of phage to target and kill their host bacterium in the context of a bacterial infection. Early work has provided insight into phage efficacy in complex living environments^6–8^. There are many known factors that influence the activity of phage *in vivo*, including route and timing of phage administration^9,10^, the host immune response including the clearance by the reticuloendothelial system^11^ and production of neutralizing antibodies, although the presence of such antibodies does not necessarily exclude a positive outcome during phage therapy^12–14^. More recent studies have shown that phage can activate the production of both pro- and anti-inflammatory cytokines and that innate immune components of the blood, particularly macrophages, may decrease the phage half-life^15,16^. Phages can be evolved in experimentally feasible time frames to overcome some of these limitations, including the enrichment of long-lasting phages with increased circulation times or the conjugation of the phage to protective carriers^17,18^. Others have sought ways to mathematically model *in vivo* phage efficacy^6,15,19,20^. Because a greater understanding of the mammalian factors that alter the effectiveness of phage would enhance our ability to improve them as targeted therapeutics, we studied the ability of phage to kill pandemic multidrug-resistant extraintestinal pathogenic *Escherichia coli* (ExPEC) in a modified form of human whole blood. Results presented here suggest a prominent role for metals in this process.

## Results

### Human blood as growth and test medium

**Figure 1:**
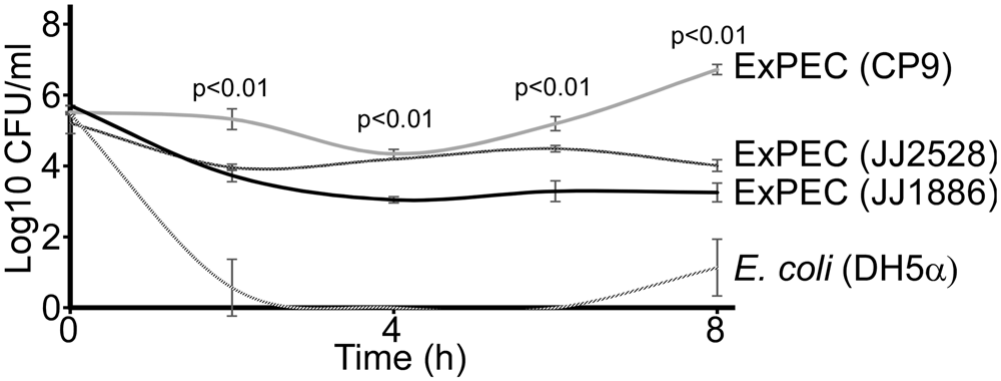
Growth of *E*. *coli* in whole human blood. ExPEC strains CP9, JJ1886, and JJ2528 as well as the commonly used laboratory strain of *E*. *coli* DH5α, were inoculated in whole human blood and incubated with shaking at 37 °C for 8 hours. Bacterial growth, reported as the logarithmic value of the number of colony forming units (CFU) per milliliter (mL) of blood, was measured at two hour intervals. The data represent the mean and standard deviation of three independent replicates.

**Figure 2:**
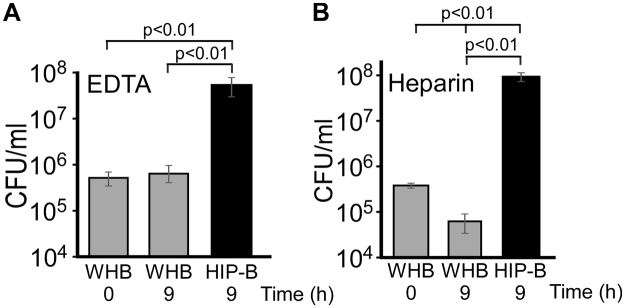
Heat inactivated plasma (HIP-B) blood as a growth medium. Whole human blood (WHB) was left untreated or treated to inactivate complement (HIP-B, see Experimental Procedures). The blood was previously treated with either the anti-coagulant EDTA (**A**) or heparin (**B**) and inoculated with ExPEC strain JJ2528 and growth measured at 9 hours as described in the legend to Fig. 1. The data represent the mean and standard deviation of three independent replicates. *p* values were determined by a Student’s t test.

Because any pharmacologic agent designed to treat a disease or condition will have its activity influenced by the intrinsic properties of blood, we sought to test the killing efficiency of phage towards *E*. *coli* in this environment. *E*. *coli* was chosen as the bacterial pathogen because it is a top cause of human bacteremia (bloodstream infections)^21–23^ and a clonal lineage (sequence type 131), harboring both multidrug-resistance (MDR) and a deadly complement of virulence factors, has become pandemic to six continents^24–26^. We first assessed the growth in whole human blood of two contemporary ExPEC strains (JJ1886 and 2528, clinical isolates from clonal group ST131)^27^ as well as a non-contemporary ExPEC strain termed CP9^28^, which is commonly used to study EXPEC virulence. *Escherichia coli* K12 (DH5α) was also tested and represents a laboratory strain commonly used for molecular biology work. Strains were seeded into whole human blood and growth monitored by recording the number of colony forming units (log10 CFU) over eight hours (Fig. 1). Unexpectedly, all three strains decreased in cell number by four hours, with the greatest effect observed for the laboratory bacterium *E*. *coli* K12. Both ST131 strains failed to recover from this attrition, whereas strain CP9 rebounded in cell numbers by six hours. The intrinsic antibacterial activity of human whole blood, therefore, may represent a complicating factor in the assessment of the lytic activity of a phage under these conditions. Hypothesizing that the negative effect on ExPEC growth in blood was due to complement, the dominant innate antibacterial mechanism in serum^29^, we sought to generate a blood formulation that preserves the cellular components of blood but eliminates the activity of complement. To do this, whole blood was first centrifuged to separate plasma (no cells present) from white blood cells and erythrocytes. The plasma was then incubated for one hour at 56 °C, a treatment that is known to inactivate complement^30^. After one hour, the plasma was then added back to the cellular fraction to form reconstituted but heat-inactivated plasma blood (or HIP-B; Supplementary Fig. 1). When ExPEC is grown in HIP-B an increase in the growth is observed at nine hours post inoculation when compared to blood that was not heated and recombined (Fig. 2A,B, *black versus grey bars*). The growth bump occurred regardless of whether the anti-coagulate EDTA or heparin were used, where 2 (EDTA) or 3 (heparin) log increases were seen when compared to untreated whole blood. This data suggests complement is a barrier to the growth of these strains and that heat inactivation of plasma and reconstitution with cells (HIP-B) facilitates the robust growth of this pathogen.

### The effect of human blood on phage killing activity

**Figure 3:**
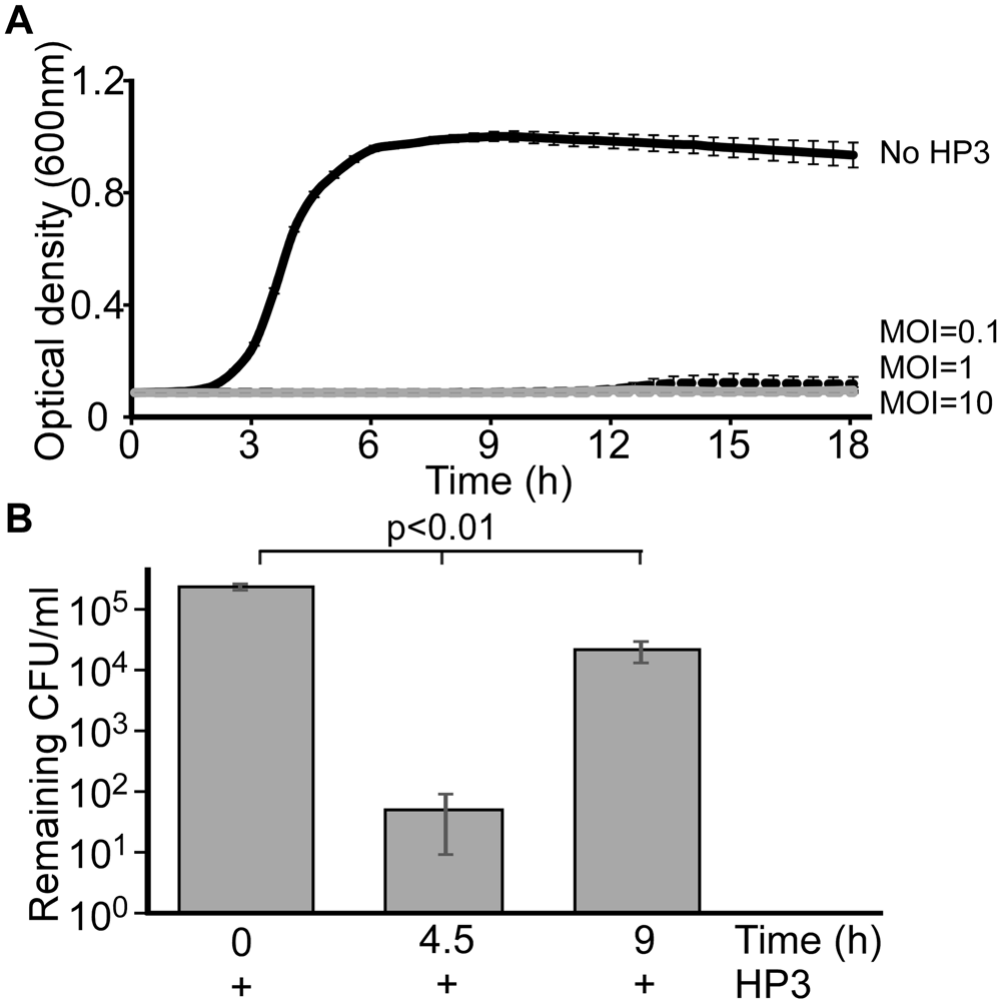
Bacteriophage treatment of cultured ExPEC. (**A**) ExPEC strain JJ2528 was cultured in L broth in the presence or absence of phage HP3 [multiplicity of infection (MOI) of 0.1, 1, and 10] and growth recorded by measuring the optical density (OD) at 600 nm every 30 minutes. (**B**) ExPEC strain JJ2528 was cultured in HIP-B in the presence of phage HP3 (MOI = 1) and growth recorded at 0, 4.5, or 9 hours as described in Fig. 1. The data represent the mean and standard deviation of three independent replicates. *p* values were determined by the Student’s t test.

Having generated a blood-based medium that facilitates the growth of ExPEC, we next assessed the ability of bacteriophage to infect, lyse and ultimately kill ExPEC under these conditions. For this purpose, we used phage HP3, which was previously isolated from goose and duck feces and displays efficacy in reducing the infectious burden of ExPEC in murine models of infection^2^. We first determined the optimal point in time during the growth of the culture to determine the effect of phage on bacterial levels. ExPEC strain JJ2528 was used because it is highly susceptible to efficient killing with the phage in LB broth, as determined by the optical density of the culture, even at relatively low multiplicity of infection (MOI - Fig. 3A). When bacterial numbers are assessed by plating a sample of the culture and counting the number of colonies (a more sensitive method than optical absorption), an approximately 3.5 logarithmic drop in CFUs is observed when ExPEC is incubated with phage in L broth (MOI of 1 for 4.5 hours - Fig. 3B). Interestingly, a rebound in the levels of ExPEC is observed at nine hours under these conditions but is still well below the approximately 10^5^ cell density reached in the absence of phage (Fig. 2). Of importance to the question of the timing for the evaluation of phage activity, this rebound effect was not seen prior to 4.5 hours, which indicates that this length of time provides a rational window in which to assess the efficiency of phage under various experimental conditions.

**Figure 4:**
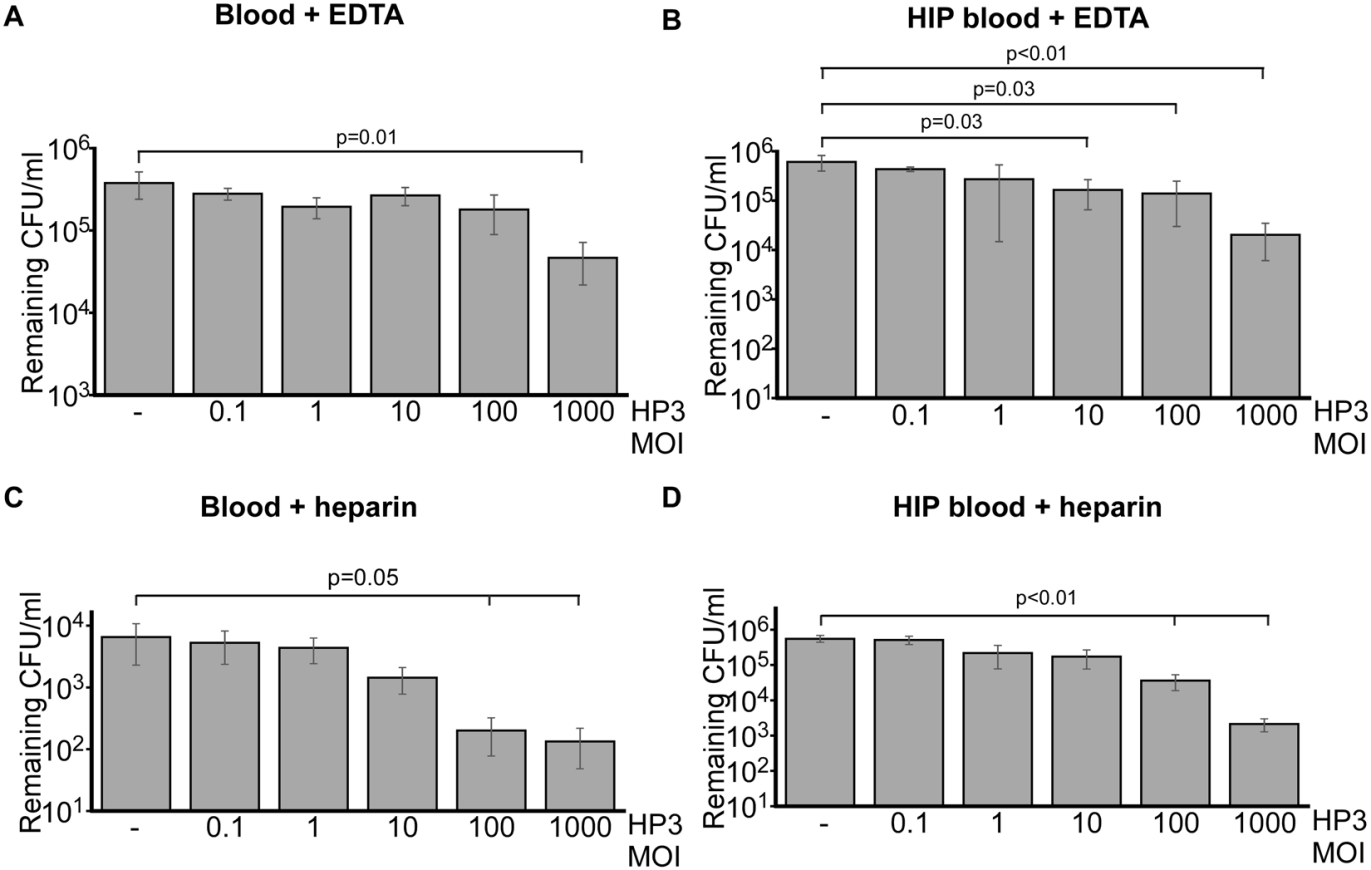
Phage killing of ExPEC in human blood. ExPEC strain JJ2528 was cultured in untreated human blood (**A** and **C**) or HIP-Blood (**B** and **D**) in the presence or absence of phage HP3 (MOI = 0.1, 1, 10, 100, or 1,000). The blood in A and B was treated with EDTA whereas the blood in C and D was treated with heparin. Bacterial growth was quantified at the 4.5 hour time point as described in Fig. 3. The data represent the mean and standard deviation of three independent replicates. *p* values were determined by the Student’s t test.

Having established these conditions in laboratory media, we next assessed the ability of ϕHP3 to kill ExPEC in human blood. ExPEC strain JJ2528 was incubated in the absence or presence (MOI 0.1–1,000) of ϕHP3 in untreated human blood or HIP blood, and the extent of killing was assessed by determining the CFUs remaining after treatment. Interestingly, unlike the very efficient 2.5 logarithmic reduction in ExPEC levels by ϕHP3 at an MOI of 0.1 in LB (Fig. 3), no decrease in bacterial cell numbers was observed under identical conditions in blood, regardless of the presence of active complement (Fig. 4A and B). Efficient phage killing activity was not observed with MOIs as high as 100; and only at an MOI of 1,000 was any significant reduction in ExPEC levels observed in HIP-B under these conditions. Hypothesizing that the anticoagulant EDTA, a metal chelation agent, might be contributing to this dramatic reduction in phage lytic activity, the same experiments were performed in untreated blood or HIP-B blood where heparin was used as the anticoagulant. Interestingly, although the phage killing efficiency was still markedly reduced compared to L broth (approximately 2–3 logarithms less efficient; compare Fig. 4C,D to Fig. 3), the killing was still 2 logarithms more efficient in blood treated with heparin than EDTA (compare Fig. 4C,D with A,B). Taken together, this data suggests the phage HP3 is 100-fold less efficient in killing ExPEC in heparized blood than it is in broth culture, which is further inhibited another 100-fold if EDTA is used as the anti-coagulant. Blood from two additional donors treated with EDTA did not yield phage-dependent killing of ExPEC in HIP-B from either donor, suggesting this effect is not due to differences in donor phenotype (Supplementary Fig. 2). Taken together, this data suggests there are cation-dependent and cation-independent factors in blood that dramatically influence the ability of phage to infect its host.

### Metal-dependent phage killing in human blood

**Figure 5:**
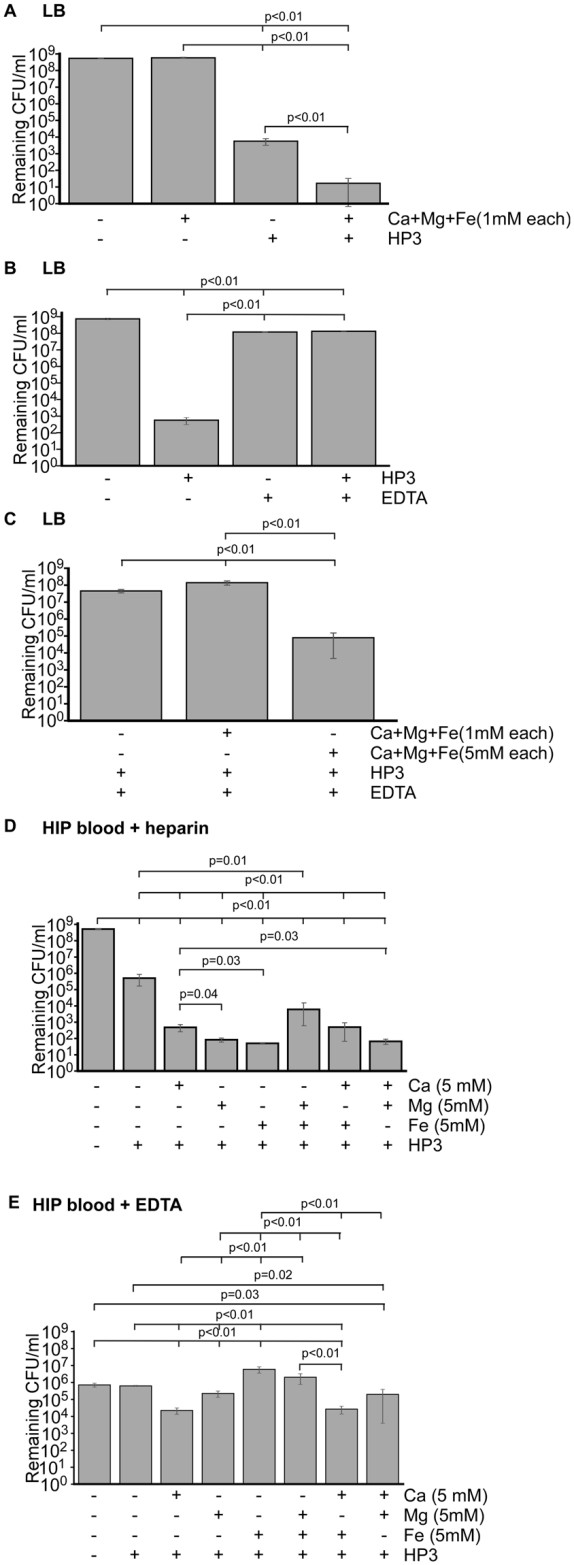
Divalent cations stimulate bacteriophage HP3 killing in blood. ExPEC strain JJ2528 was grown in LB (**A**–**C**) or HIP-B (**D**,**E**) in the presence of EDTA (4 mM), HP3 (MOI = 1), or divalent metals (1 or 5 mM) where indicated. The levels of ExPEC were determined by plating the cultures at the 4.5 hr point (optimal for phage killing) and recording the number of colony forming units per milliter of culture. Data shown are one representative biological replicate of three independent experiments which each had at least three technical representatives. *p* values were determined by a Student’s t test.

The lack of efficient killing by phage in heat-treated blood with EDTA led us to hypothesize divalent cations may be important for phage activity in this environment. To test the hypothesis that metals are important in this context, we assessed the contribution of three of the most prominent metals present in blood (Ca^2+^, Mg^2+^, and Fe^2+^) to phage activity towards ExPEC. This effect was first studied in L broth. Whereas the addition of phage resulted in a 5-log reduction in bacterial levels, this was further improved by another 2-logs when a cocktail of calcium, magnesium, and iron was added (Fig. 5A), demonstrating metals can have a stimulatory effect in a medium already rich in such factors. When the converse experiment was conducted and the levels of free metals were chelated by EDTA, a greater than 5-log inhibition in phage killing was observed (Fig. 5B). This inhibition was overcome by the addition of exogenous metals when the concentration of the metals was 5 times that over the concentration of EDTA (5 mM), but not at equimolar with EDTA (1 mM, Fig. 5C). These findings indicated that low availability of free metal cations could be the reason for the loss of phage activity in human blood. To test this idea, HIP-blood anticoagulated with heparin was treated with phage (MOI of 1). Calcium, magnesium, and iron, added individually or in pairs, stimulated phage-dependent killing of bacteria approximately 10,000-fold, regardless of the metal added (Fig. 5D). In addition, EDTA-dependent inhibition of phage killing of ExPEC was overcome by the addition of calcium (~ 1.5 log improvement) and magnesium (~0.5 log improvement) but not by iron, which dramatically stimulated the growth of ExPEC (Fig. 5E). Cocktails of calcium/iron and calcium/magnesium also stimulated phage killing, but a cocktail of magnesium/iron, did not (Fig. 5D,E). The addition of iron likely aids bacterial growth by overcoming the known host-driven chelation of free iron in blood^31^. Taken together, there data strongly suggest that these metals are limiting factors in blood that influence the ability of phage to infect their host bacterium.

### Metal dependent killing by phage in blood applies to several ST131

**Figure 6:**
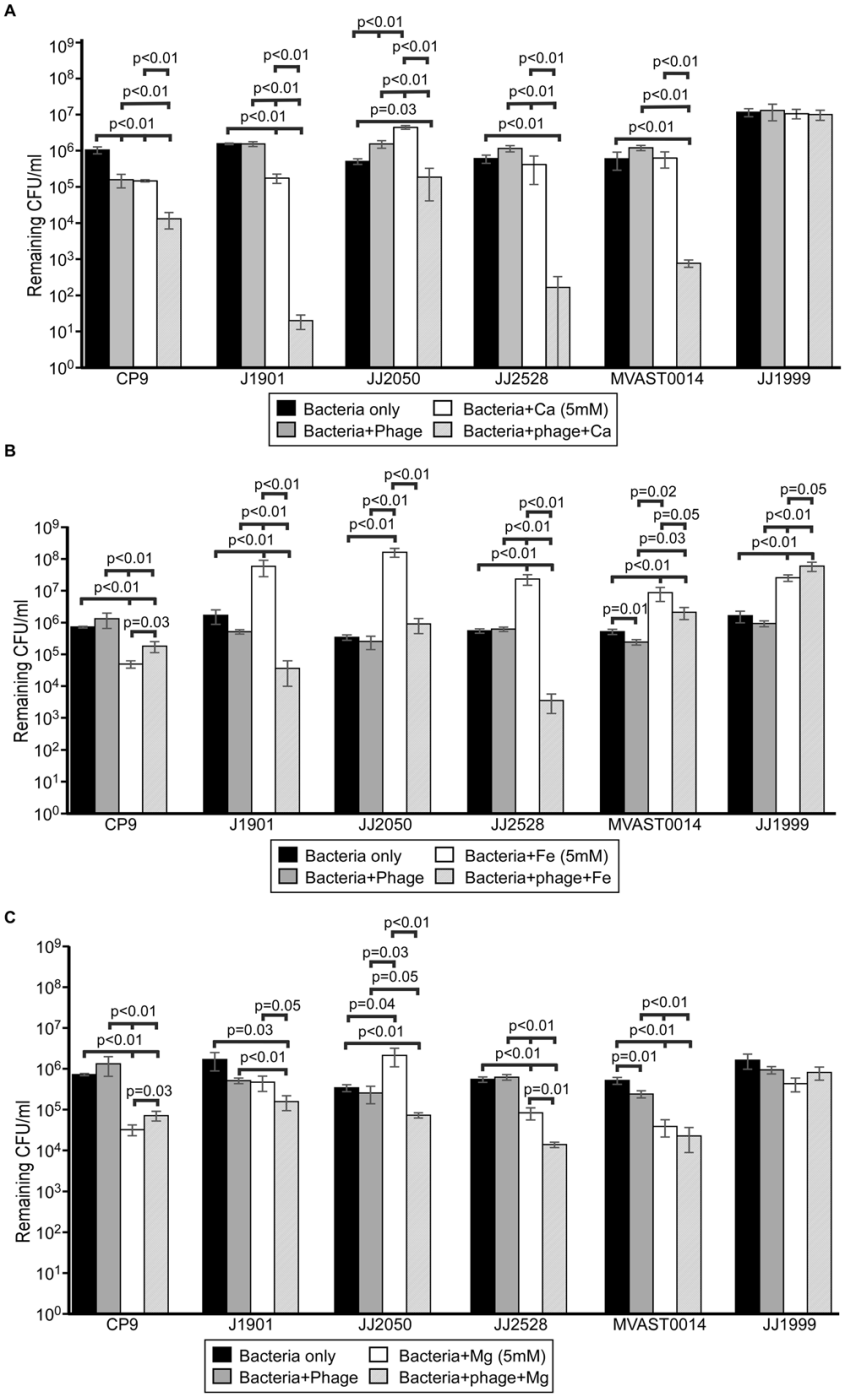
Effect of cations on bacteriophage killing of pandemic ExPEC strains. ExPEC strains JJ1901, JJ1999, JJ2050, JJ2528, and MVAST0014 were mixed with bacteriophage HP3 (MOI = 1) and grown in HIP reconstituted human blood anticoagulated with EDTA. CaCl_2_ (**A**), FeSO_4_ (**B**), or MgCl_2_ (**C**) were added at a final concentration of 5 mM. Bacterial growth was determined recording the number of colony forming units at 4.5 hours. Data represent the mean and standard deviation from three independent replicates. The *p* values were determined by Student’s t test.

We assessed the broad applicability of these findings by assessing the effect of calcium, iron, or magnesium phage activity in blood for 5 different ExPEC strains. The addition of phage had very little effect on the survival of ExPEC in blood. In 4 of 5 cases examined, the addition of calcium significantly stimulated the killing of ExPEC (range 0.5 logs to nearly 5 logs – Fig. 6A). Only JJ1999 demonstrated complete resistance to phage and phage + calcium, and for reasons currently unknown. Unlike calcium, iron induced robust growth in blood in all ST131 strains, signifying the iron starved nature of these cells under host-like conditions (Fig. 6B). Like calcium, iron stimulated the killing of ExPEC, especially if iron previously enhanced the growth of the *E*. *coli* strain, with JJ1999 the exception once again. The results were similar for magnesium, where 4 of 5 strains showed magnesium-dependent killing by phage of ExPEC (Fig. 6C).

**Figure 7:**
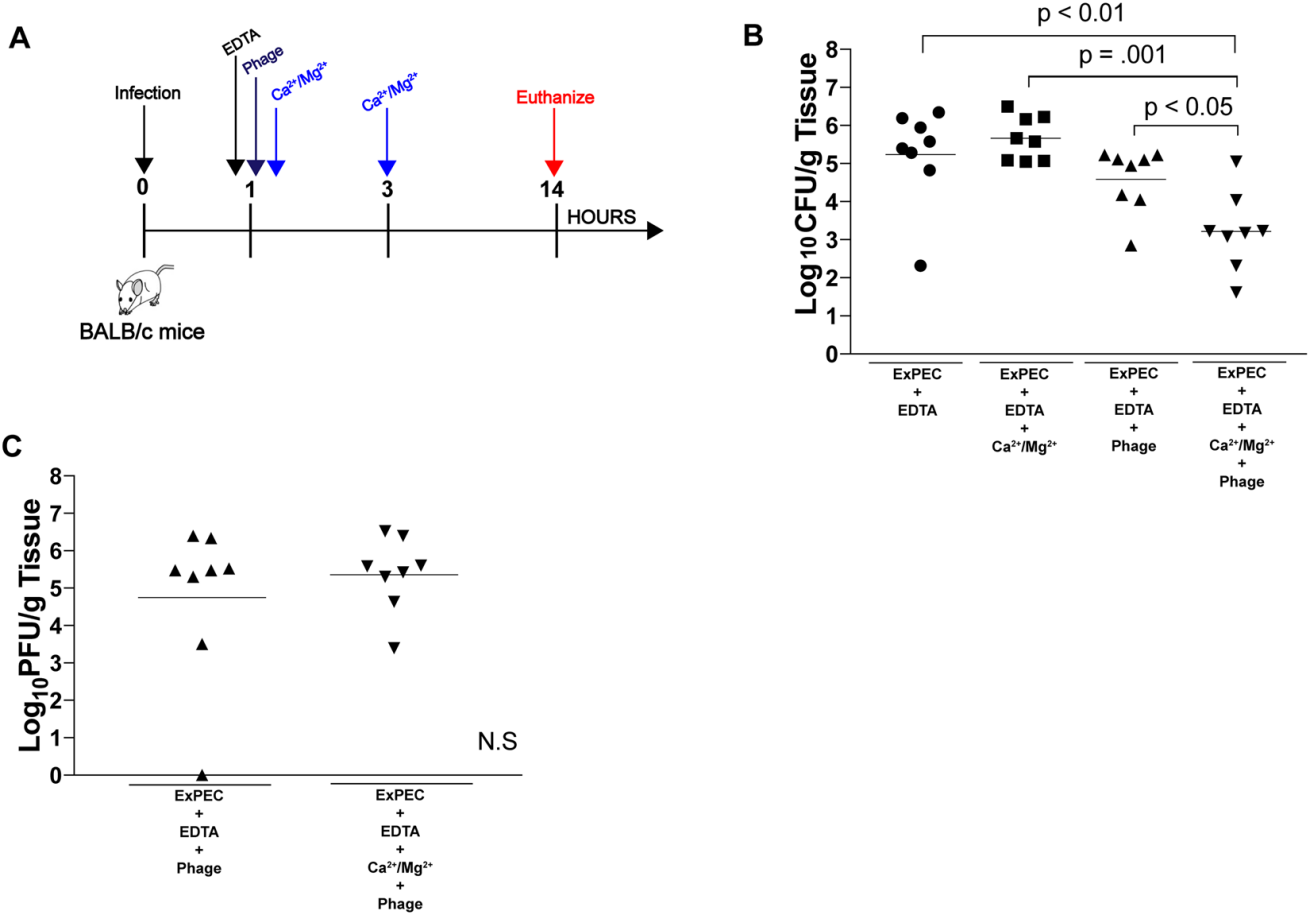
Subcutaneous ExPEC Infection (**A**) Mice were infected subcutaneously with ST131 isolate, JJ2528. One hour post-infection all mice were injected with either EDTA (4 mM), EDTA plus Ca^2+^/Mg^2+^ (5 mM), EDTA + phage (10^6^ PFU) or EDTA + Ca^2^^+^/Mg^2+^  + phage. A second dose of cations was given to mice 3 hours post-infection (all injections were given separately). (**B**) Bacterial counts in log CFU/gram of tissue at the site of infection 14 hours post-infection. (**C**) Phage counts in log PFU/gram of tissue at the site of infection. A one-way ANOVA analysis and Tukey’s test for multiple comparisons was performed on log transformed data to determine significance. *p* values are noted above the bars and n is equal to 8.

To determine if calcium and magnesium are important for the *in vivo* efficacy of these phage, we developed a murine model of localized subcutaneous ExPEC infection that develops in less than 24 hours^32^. In this model, an ExPEC strain is delivered subcutaneously and the infection followed by monitoring the levels of the bacteria in the developing abscess with or without treatment. ExPEC strain JJ2528, a clinical isolate that demonstrates metal-dependent phage sensitivity (this work), was delivered subcutaneously in BALB/c mice followed by treatment with either EDTA, EDTA plus Ca^2+^/Mg^2+^, EDTA plus phage HP3, or EDTA plus Ca^2+^/Mg^2+^ plus phage HP3. The addition of EDTA in this experiment allows us to modulate the levels of endogenous free metals which would otherwise not be feasible by other means. The levels of ExPEC in tissue were then assessed by plating tissue after necropsy on LB/agar (Fig. 7A). As observed in Fig. 7B, the addition of Ca^2+^/Mg^2+^ had little effect on the levels of ExPEC. When phage was added, the levels of ExPEC decreased but the trend was not statistically significant. Interestingly, when Ca^2+^/Mg^2+^ were added in combination with phage, a greater than 1.5 log reduction in bacterial levels were observed (p < 0.01 compared to just Ca^2+^/Mg^2+^ and p < 0.05 compared to just phage HP3). In both the phage treated groups, a significant number of phage were found in this tissue as well, with slighter higher, but not statistically significant, levels observed for the group treated with metals (Fig. 7C). This data suggests that the metals calcium and magnesium can stimulate the killing of ExPEC by phage in a murine model of *E*. *coli* bacteremia.

## Discussion

Because of the importance of metals in biology and their abundance on Planet Earth, phage have also evolved the use of metals such as iron, calcium, and magnesium. In addition to being dispersed throughout the environment, these metals are embedded in each component of bacterial cells, from cytosol to cell surface, and could thus be influential in the infection of such cells by phage. They are also found in human blood, and their circulation to cell and tissues is important in basic human health. Most studies to date have focused on the effect of metals on phage infection of their bacterial hosts grown in common laboratory media. As early as 1925, Stassano and de Beaufort recognized a requirement for divalent cations in phage activity by observing the negative effect citrate plays on phage production^33^. In 1928, Bordet and Renaux reported that the addition of calcium chloride to culture stimulated Shiga phage activity^34^. Similar results with calcium were reported for typhoid and coli phages in the early 1950s^35,36^. The importance of metals during phage infection also extends to Gram-positive bacteria. Phage MR-10, which targets methicillin resistant *S*. *aureus*, demonstrates increased phage titers in the presence of calcium^37^. Phages BCP1–1 and BCP-8–2 target bacillus species, and calcium enhances this response^38^. Delbruck performed the first mechanistic studies in 1948 of the effect of metals on infection and found that calcium stimulated the adsorption of phage to *E*. *coli*^39^. This finding led to several seminal studies that focused on how metals are important in the various stages of the infectious process. For example, low calcium induces depolarization of the bacterial cell membrane during infection by T5, thereby preventing the transfer of DNA^40^. Magnesium, in addition to calcium, stimulates steps beyond adsorption including the intracellular synthesis of phage progeny^36,41,42^. Metals are also co-factors that contribute to the maintenance of the structure of phage proteins. This is best evidenced by the tail spike protein of phage P2 whose membrane-spanning apex coordinates both iron and calcium atoms^43^. In fact, others have calculated that phage are significant chelators of oceanic metal and may use this strategy to gain access to siderophore receptors on the bacterial surface, the so-called ferrojan horse hypothesis^44^. When phage HP3 is preincubated with metals, dialyzed, and then added to HIP-blood containing ExPEC, there is no metal-dependent enhancement of killing, which seemingly precludes the ferrojan horse hypothesis as the mechanism responsible for the findings herein (data not shown).

The exact mechanism by which metals enhance the killing of ExPEC by phage is currently unknown. The metal dependent killing described herein seems to be specific to phage HP3 since two other phage, EC1 and CF2, did not demonstrate this effect on ExPEC, despite the fact that these two phages also efficiently kill this bacterium (not shown)^2^. Furthermore, only iron seemed to promote the growth of ExPEC, thereby ruling out metal-dependent growth enhancement as a primary reason for this effect. When EDTA was used to chelate the metals from purified phage preparations that were examined by cryo-electron microscopy, there was no difference in the phage structure, number of empty capsids, or orientation of the tail fibers compared to metal-replete conditions; and preincubation of the metals with phage did not increase the number of phage progeny produced, nor did it alter the burst size (not shown). However, it is interesting to note that open reading frame 3 of HP3, which is homologous to GP34 of T4 Phage and thus might encode a proximal long tail fiber, is predicted to encode a domain with four calcium binding sites (RAPTOR X structural analysis)^2,45,46^. This region is not present in related phages EC1 and CF3, which are not stimulated to kill ExPEC in a calcium-dependent manner. Such information suggests calcium enhances the interaction of phage with its host, or stabilizes the complex for DNA injection. Future studies will examine the merits of this hypothesis.

Perhaps the greatest challenge to the realization of phage therapy being approved and regularly used to treat infections in the clinics of Western society is that phage are a biologic. Biologics are defined by the U. S. Food and Drug Administration as being anything derived from biology (e.g. viruses, blood products like antibodies, proteins, etc) that may have therapeutic value in the treatment of a specific disease. The regulation and approval of such biologics is inherently more challenging than that of chemically synthesized small molecule drugs whose method of preparation, purity, and composition is defined. Biologics may harbor other components in their formulation that may have off-site effects, and the lack of control in their production (compared to strict chemical synthesis protocols) may mean the active agent has variability from preparation to preparation. The pharmacokinetics of biologics may also be more complex. A small molecule with a narrow chemical space may have a limited interaction with the multitude of tissue and physiologic processes that the body uses; biologics, because of their greater complexity, may interface with many more systems. Being a virus, an effective phage will need to avoid clearance, destruction by the innate immune system, non-specific binding to tissues, and adaptive immune responses that may neutralize activity via antibody. For example, in mice with a functional immune system, approximately 1% of the intravenously injected T7 phage are removed by B cells in 60 minutes^47^. Mononuclear phagocytes are also responsible for the rapid clearance of phage^12^. There are physical constraints because of their large size as well, which may result in poor penetrance. The intraperitoneal delivery of phage is associated with enhanced levels of phage in critical organs, and prolonged levels over time^48^. The spleen and liver seem to have a substantial effect on the clearance of phage^49,50^. The spleen in particular retains phage for nearly two weeks after administration into rabbits^51^. Other routes of phage administration have shown promise, but results vary widely. Intravenous injection allows phage to spread rapidly to major organ systems^52,53^. When phage are administered intranasally to mice, they reach the blood^52,54,55^. Intramuscular administration of phage is efficacious in animals^52,54,55^. The adaptive immune response leads to the production of antibodies to phage, including IgA, IgG, and IgM when phage were delivered orally and IgG and IgM when phage are delivered intraperitoneally^14,15,56^. In some cases, the antibodies are neutralizing and hence reduce phage efficacy^57,58^. In addition to the above-mentioned physical and cellular factors that may be barriers to *in vivo* lytic activity, the data described in this report suggests prominent blood components, even essential metals, may influence phage activity. This means that in addition to immune components, one must also consider other components of blood that are distinct from the immune system. Although metals may have a role as defined here, we might also consider ingested xenobiotics, hormones, carbohydrates, lipids and other small metabolites in possibly altering phage activity. The generation of HIP blood, as reported here, may be a useful surrogate to determine how blood factors influence phage efficacy; or provide a useful screening tool on an individual patient basis that allows one to pretest the activity of a phage on a target bacterial pathogen prior to administration during an actual treatment. It may thus be possible to significantly enhance the killing power of phage by fine tuning their metal sensitivity, or use phage that are metal-independent during therapy. All of these approaches may not only inform us on the selection of phage likely to be efficacious, they also may help precisely define guidelines or treatment regimens that are more likely to be successful.

To summarize, we report here that (i) heat-inactivation of plasma and reconstitution with red/white blood cells makes a useful blood-based “medium” to study the factors that affect the killing of ExPEC by phage; (ii) that lytic phage that efficiently infect and kill ExPEC in L broth require 4 orders of magnitude more phage for an equivalent level of killing in human blood; (iii) that at least 2 orders of this phage inefficiency in human blood is due to the anti-coagulant EDTA, a potent scavenger of divalent cations; (iv) that the metals calcium, magnesium, and to some degree iron are necessary for efficient phage killing activity towards ExPEC in blood and; (v) that these metals are important for phage to kill other strains of ExPEC under similar conditions. These results suggest it may be beneficial to isolate, select, or engineer therapeutic phages that are metal-independent, or, at the very least, if the phage requires metals for full activity, to administer the relevant metal at the time of administration.

## Materials and Methods

### Bacterial strains, phage and materials

Bacterial strains used in this study include extraintestinal pathogenic *E*. *coli* strains CP9, JJ1886, JJ1901, JJ1999, JJ2050, JJ2528, and MVAST0014, which were generously provided by James Johnson (University of Minnesota)^24,59^. The metal salts CaCl_2_, FeSO_4_, and MgCl_2_ were purchased from VWR International (Radnor, PA). Bacteriophages HP3 (accession number KY608967), EC1 (accession number KY608965, and CF2 (accession number KY608966) were isolated and purified as previously described^2^.

### Heat-inactivated Plasma Blood (HIP-B)

Human blood was either purchased from the commercial vendor Innovative Research (Novi, MI) or attained from the Houston Blood Bank. There are no institutional regulations regarding the use of blood from these sources. No human subjects were used in this research. The blood was treated either with EDTA (1–2 mg/mL) or heparin (0.2 mg/mL) where indicated. Heat-inactivated plasma blood (HIP-B) was generated by first centrifuging whole human blood at 2000 × g for 10 minutes and subjecting the plasma fraction to 56 °C for 1 hour, a step known to inactivate the complement system^30^. The treated plasma was then added back to the pelleted red and white blood cell fraction to reconstitute the blood with all components, minus complement (Supplementary Fig. 1).

### Bacteriophage purification and titer

The purification and titer of all phages was determined as previously described^60^. Briefly, phages were serially diluted and 5 µl of each diluent was spotted onto LB top agar (0.75%) pre-mixed with 100 µl of an overnight culture of JJ2528. After incubation at 37 °C for 18 hours, the number of plaques on each plate for each dilution were recorded and the titer calculated. Phages were stored at 4 °C in phage buffer until use.

### Bacterial growth and survival assays

All bacterial growth assays were performed in 96-well microtiter plates with 200 µl of LB and a starting optical density at 600 nm (OD_600_) of 0.001. The cultures were then maintained under continuous shaking for 18 h at 37 °C with optical measurements at OD_600_ recorded with a BioTek synergy HT plate reader every 30 minutes. For the bacterial survival assays in LB or HIP-B, *E*. *coli* was cultured as above and phage HP3 added at a multiplicity of infection (MOI) of 1 or 10. For the bacteria + phage + cation samples, CaCl_2,_ FeSO_4_, or MgCl_2_ were added at a final concentration indicated in the figure legend, followed with the addition of phage HP3 (MOI = 1). Optimal metal-dependent killing by phage HP3 was observed between 1 and 5 mM (final concentration) of metals. The cultures were then incubated at 37 °C with continuous shaking for either 1 or 4.5 hours, at which point each sample was assessed for the number of colony forming units (CFUs) by 10-fold serial dilution of the cultures.

### Subcutaneous Mouse Infection

Baylor College of Medicine’s Institutional Animal Use and Care Committee (IACUC) approved the murine infection studies presented in this work (approval number AN5177) and all methods were carried out in accordance with relevant guidelines and regulations.

Groups of male and female BALB/c mice (Jackson Laboratories, Bar Harbor, ME) ranging in age from 10 to 12 weeks were used for this study. All mice received food and water ad libitum. All methods performed on mice were approved by our Institutional Animal Care and Use of Committee (IACUC) and were determined to be in accordance with “The Guide and Care and Use of Laboratory Animals” (National Institute of Health).

Mice received 10^7^ CFU of ST131 clinical isolate JJ2528, a clinical isolate shown to be virulent in an intraperitoneal mouse model of infection and sensitive to phage HP3 *in vivo*^2^. Mice were injected subcutaneously at the nape of the neck with JJ2528, which was previously shown with to create a localized granuloma-like infection at the site of injection^32^. One hour following injection, mice were injected with 10^6^ PFU of purified phage HP3, Ethylenediaminetetraacetic acid (EDTA; 4 mM; Fisher, BP118.500) and CaCl_2_ and MgCl_2_ (5 mM each), individually and in combination (see Fig. 7). A second dose of cations (CaCl_2_ and MgCl_2_) at 5 mM each was given 3 hours post-infection. After 14 hours mice were euthanized, necropsied and at the site of infection underneath the fur was excised and plated to quantify bacteria and phage levels, represented as CFU or PFU per gram of tissue, respectively.

### Statistics

All data represent the mean and standard deviation of at least three technical replicates, each of which is representative of one of three independent biological replicates. A One-Way ANOVA test was performed to determine the significance of multi-group data in all figures and significance (*P* value) was determined by a Student’s *t* test to compare two sets of data within the group for Figs 2–6 and Supplementary Fig. 2.

## Electronic supplementary material

Supplementary Information
