# Supplementary material for: Metals Enhance the Killing of Bacteria by Bacteriophage in Human Blood
Source: Sci Rep. 2018 Feb 2;8:2326. doi: 10.1038/s41598-018-20698-2 (PMC5797145; doi:10.1038/s41598-018-20698-2)
Supplement: Supplementary file 1 — Supplementary Information [file 41598_2018_20698_MOESM1_ESM.pdf]

Metals Enhance the Killing of Bacteria by Bacteriophage in Human Blood

Li Ma[1,4], Sabrina I. Green[1,4], Barbara W. Trautner[2,3], Robert F. Ramig[1], and Anthony W. Maresso[1*]

[1]Molecular Virology and Microbiology Department, Baylor College of Medicine, Houston, TX

[2]Michael E. Debakey Veterans Affairs Medical Center, Houston, TX

[3]Department of Medicine, Baylor College of Medicine, Houston, TX

[4]Contributed equally

* Correspondence to maresso@bcm.edu

Key words: *Escherichia coli*, ExPEC, cations, blood, bacteriophage, therapy

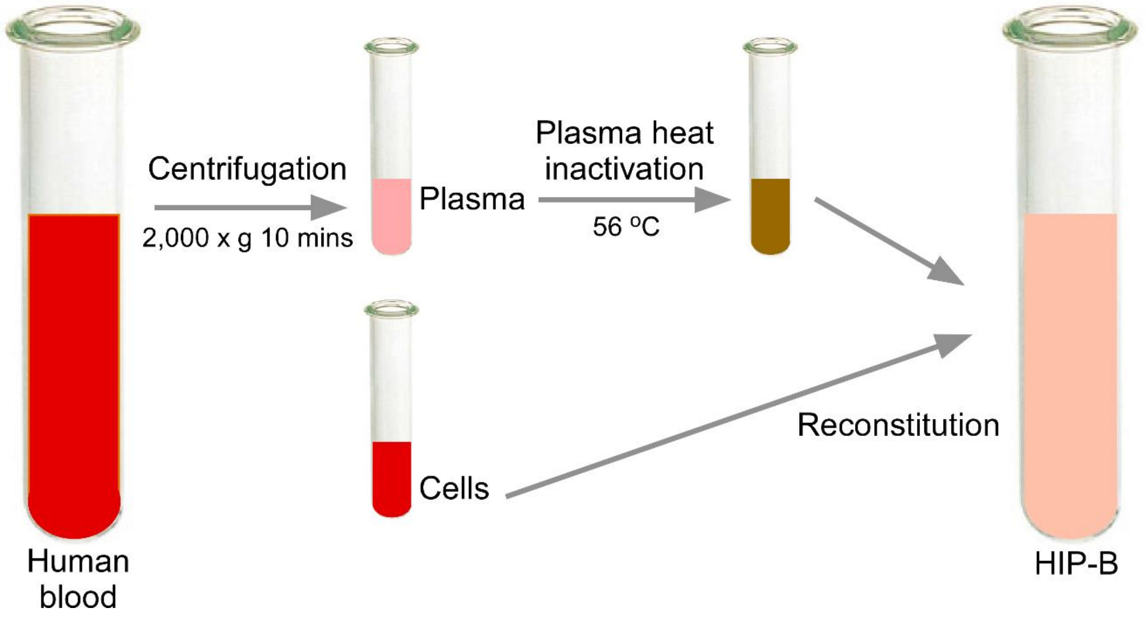

Supplement Figure 1. Preparation of HIP-B. Human blood was centrifuged at 2000 X g for 10 min and the supernatant (the plasma portion) was subjected to 56°C for 1 hour. Immediately after heat inactivation, the cell pellet portion was mixed with the supernatant to constitute the HIP-B.

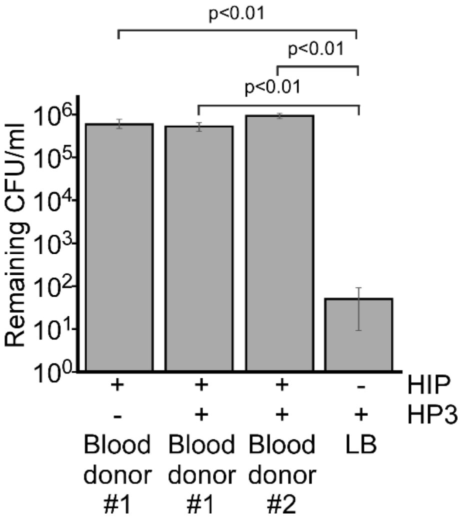

Supplement Figure 2. The effect of EDTA on bacteriophage lytic activity in HIP human blood. ExPEC strain JJ2528 was grown in HIP reconstituted human blood anticoagulated with EDTA with or without bacteriophage HP3 (MOI=1). Bacterial growth was determined by recording the levels of colony forming units after 4.5 hours. Data represent the mean and standard deviation from three independent replicates. The *p* values were determined by Student's t test.
